# Improving efficacy of TNBC immunotherapy: based on analysis and subtyping of immune microenvironment

**DOI:** 10.3389/fimmu.2024.1441667

**Published:** 2024-10-04

**Authors:** Yalan Yang, Haifeng Li, Wei Yang, Yanxia Shi

**Affiliations:** ^1^ State Key Laboratory of Oncology in South China, Guangdong Provincial Clinical Research Center for Cancer, Sun Yat-sen University Cancer Center, Guangzhou, China; ^2^ Department of Medical Oncology, Sun Yat-sen University Cancer Center, Guangzhou, China

**Keywords:** immunotherapy, breast cancer, microenvironment, TNBC, immune subtype, therapeutic target, biomarker

## Abstract

Triple-negative breast cancer (TNBC) is a highly aggressive type of breast cancer that encompasses several distinct subtypes. Recent advances in immunotherapy offer a promising future for the treatment of these highly heterogeneous and readily metastatic tumors. Despite advancements, the efficacy of immunotherapy remains limited as shown by unimproved efficacy of PD-L1 biomarker and limited patient benefit. To enhance the effectiveness of TNBC immunotherapy, we conducted investigation on the microenvironment, and corresponding therapeutic interventions of TNBC and recommended further investigation into the identification of additional biomarkers that can facilitate the subtyping of TNBC for more targeted therapeutic approaches. TNBC is a highly aggressive subtype with dismal long-term survival due to the lack of opportunities for traditional endocrine and targeted therapies. Recent advances in immunotherapy have shown promise, but response rates can be limited due to the heterogeneous tumor microenvironments and developed therapy resistance, especially in metastatic cases. In this review, we will investigate the tumor microenvironment of TNBC and corresponding therapeutic interventions. We will summarize current subtyping strategies and available biomarkers for TNBC immunotherapy, with a particular emphasis on the need for further research to identify additional prognostic markers and refine tailored therapies for specific TNBC subtypes. These efforts aim to improve treatment sensitivity and ultimately enhance survival outcomes for advanced-stage TNBC patients.

## 1 Introduction

Triple-negative breast cancer is a highly aggressive and heterogeneous type of breast cancer (BC) that lacks the expression of estrogen receptor (ER), progesterone receptor (PR), and human epidermal growth factor receptor 2 (HER2). These features make TNBC non-responsive to conventional hormonal and targeted therapies, resulting in poor clinical outcomes (1, 2).

Immunotherapy (IM) emerges as a promising therapeutic strategy for TNBC by leveraging immune system to identify and eradicate tumor cells. Several clinical trials have demonstrated the potential benefits of IM for TNBC patients, especially when combined with chemotherapy (3, 4, 5–8). In neoadjuvant setting, IMpassion031 showed that adding atezolizumab, an anti-PD-L1 antibody, to chemotherapy increased the pathological complete response (pCR) rate from 41.1% to 57.6% in patients with early-stage TNBC (9). In the metastatic setting, KEYNOTE-355 demonstrated that combining pembrolizumab, an anti-PD-1 antibody, with chemotherapy improved the overall survival (OS) from 16.1 months to 23.0 months in patients with PD-L1-positive TNBC (10). Based on these results, the FDA approved atezolizumab plus nab-paclitaxel and pembrolizumab plus chemotherapy for patients with early-stage and metastatic TNBC patients respectively with PD-L1 expression of 1% or more (11).

However, the effectiveness of immunotherapy for TNBC is not universally established, as evidenced by the IMpassion131 trial, which failed to show any benefit of adding atezolizumab to paclitaxel in patients with metastatic TNBC (12). The variation in chemotherapy choice between IMpassion131 and Impassion130 led to significant differences in trial results, indicating uncertainty in the effectiveness of immunotherapy for metastatic tumors. One of the major challenges in optimizing IM for metastatic tumors is the heterogeneity of the tumor microenvironment (TME). The TME in TNBC consists of various immune and stromal cells that can either promote or inhibit tumor growth and response to therapy. The complex interactions within the TME, including the presence of immune cells such as tumor-infiltrating lymphocytes (TILs) and the expression of immune checkpoint molecules like PD-1, LAG-3, and IDO, play crucial roles in determining the response to immunotherapy (11, 13).

The identification of reliable biomarkers for evaluating IM response and predicting resistance remains a significant challenge. Although PD-L1 expression is currently used as a biomarker for IM selection, it has several limitations, such as variable expression patterns, low specificity, and dynamic changes during treatment (14). Therefore, comprehensive and robust biomarkers are needed to capture the complexity and diversity of TNBC, thus providing a basis for patient identification and stratification treatments.

TNBC is not a single entity, but rather an umbrella that encompasses various subtypes with distinct genetic, transcriptional, histological, and clinical characteristics (15). These subtypes may have different immune phenotypes and responses to IM. Therefore, understanding the subtyping of TNBC based on TME features may help to improve patient stratification and tailor IM accordingly. In the present study, we review the current knowledge on the TME characteristics and subtyping of TNBC and discuss how they can be used to guide IM selection and overcome resistance.

## 2 The heterogeneity of tumor microenvironment in TNBC

**Figure 1:**
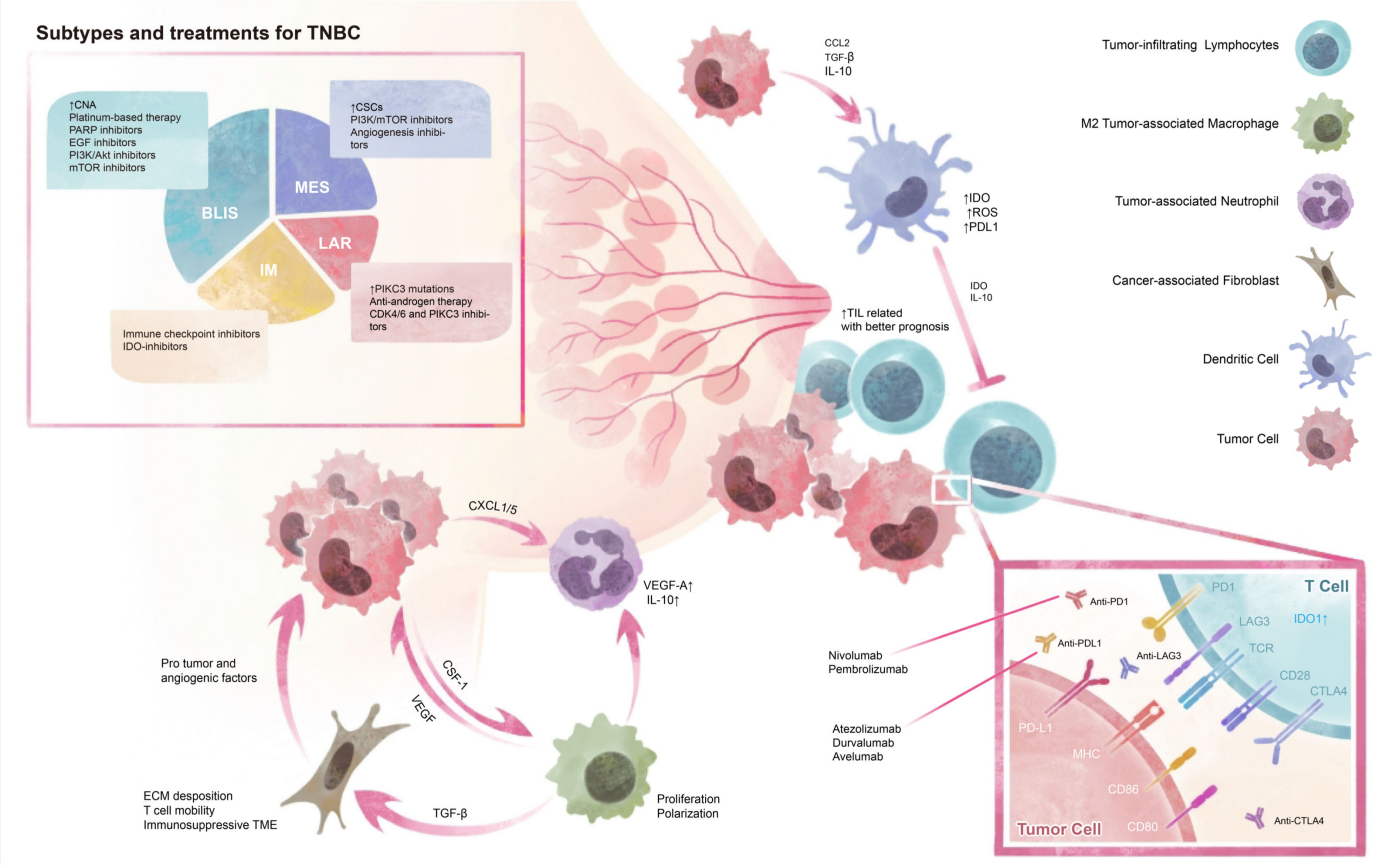
Tumor Microenvironment and Subtypes of TNBC. TNBC can be classified into four subtypes based on their genetic profiles and microenvironment, which provide diverse therapy choices. The tumor environment contains cellular interactions and molecular modulations. CNA, copy number alternations; CSCs, cancer stem cells; BLIS, basal-like immunosuppressed; MES, mesenchymal; LAR, luminal androgen receptor; IM, immunomodulatory.

The tumor immune microenvironment is featured by the neoplastic growth region along with the extracellular matrix and other anatomical constituents (16). The tumor microenvironment is a crucial factor in the progression of TNBC as well as its response to therapy, which exhibits significant inter-patient variability and is closely associated with treatment prognosis (**Figure 1**). Identifying spatial immune biomarkers can help to differentiate intrinsic prognostic immune features and inform therapeutic strategies for clinically actionable immune biomarkers in TNBC. At present, molecular components especially PD-1/PD-L1 expression are identified as first-line biomarkers for recognizing patients responsive to immune checkpoint blockade (ICB). However, the cellular components, namely stromal cells (fibroblasts, macrophages, and endothelial cells) and immune cells (T lymphocytes, etc.) also play a crucial role in the effect of TME on tumor progression and have gained increasing attention in the investigation of tumor immune landscape (17). The variation in cellular components significantly influences the therapeutic outcomes and underscores the importance of a comprehensive understanding of the TME. Furthermore, gene signatures corresponding with different features or cellular components can identify extracellular components such as growth factors, cytokines, hormones, extracellular matrix, and molecular component markers, thus having potential clinical effects (17, 126). This heterogeneity necessitates the integration of various biomarkers and gene signatures to develop more precise and personalized treatment strategies. Understanding the dynamic interactions within the TME is essential for the successful application of immunotherapies and improving patient outcomes in TNBC.

### 2.1 Cellular components of TME

Since 2014, The collection of TILs data has been conducted on a global scale, encompassing over 20,000 primary breast cancer samples (18, 19). The findings have reinforced a strong correlation between improved prognoses and the presence of TILs in both TNBC and HER2+ BC (20, 21). TILs therefore have been widely acknowledged as a well-known prognostic factor in early triple-negative breast cancer. The KEYNOTE-086 study found that patients with more TILs were inclined to get better results from ICB treatment (22, 23). A recent study from Nature also reinforces that TILs have an independent association with TNBC prognosis (16). A comprehensive evaluation of TME identified two subtypes correlated with TILs levels and immune-related pathways (24), among which the IM TNBC subtype was associated with better prognosis and response to chemotherapy and immune therapy in both the neoadjuvant and adjuvant cohorts (15, 25). To investigate the potential correlation between increased TILs and specific T cell subtypes, an immunophenotypic characterization has been conducted (26). This analysis revealed a consistent positive association between the overall number of TILs and all T cell subtypes, particularly emphasizing the density of CD8+ T cells, CD4+ lymphocytes, and FOXP3-expressing cells. High levels of TILs were found to be positively correlated with prolonged relapse-free survival and overall survival in TNBC patients (27, 28). Therefore, TILs could be identified as a subtyping biomarker for immune response TNBC. Neoplasms have demonstrated the ability to elude immune system defenses through a variety of mechanisms, including antigen recognition constraints, immune system suppression, and T cell exhaustion induction (29). By receiving personalized TILs immunotherapy, patients with refractory solid tumors, including TNBC, could have a better prognosis (30, 31). In 2018, Zacharakis reported a chemotherapy-resistant HR+ metastases BC patient who received TILs reactive against four proteins achieved a durable complete response after combination therapy with IL-2 and ICB (30). In 2020, preliminary efficacy for the combination of PD-1 inhibitor and TILs therapy in NSCLC treatment was reported (32). Despite promising results from clinical trials, there remain substantial challenges in broadening the application of TILs immunotherapy. The unknown characteristics of the high heterogeneity of TILs, immune evasion mechanisms, and limited patient response need to be addressed in future research agendas. In conclusion, TILs immunotherapy may provide a very promising treatment method for patients with drug-resistant TNBC.

Moreover, a population of macrophages in TNBC microenvironment suggests a better prognosis for immunotherapy. Tumor-associated macrophages (TAMs) represent a diverse population characterized by pronounced plasticity and have been linked to tumor aggressiveness and unfavorable prognostic outcomes. M2 polarization of macrophages is one of the major reasons for immunosuppression (33), while macrophage-enriched subtype (MES) of TNBC displays responses to ICB (34, 35). However, macrophages expressing CD206 demarcate a subgroup of TNBCs that may have a better prognosis (36). Therefore, the relationship between macrophages and patient prognosis in TNBC is complex and may depend on the specific macrophage subtype. Challenges in targeting TAMs include overcoming their plasticity and immunosuppressive functions, as well as ensuring specificity to avoid adverse effects on normal macrophages. Macrophages-enriched subtype transit to neutrophils-enriched subtype (NES) when tumor develops resistance to ICIs, which contributes to immune suppression (35). Tumor-associated neutrophils (TANs) are recruited to the tumor site by tumor-derived factors and can also be further classified into N1 and N2 subtypes based on gene expression profiles despite the lack of markers to differentiate. TANs have pro-tumor activity by producing ARG1, IL-10, TGF-β, and VEGF, and inhibiting cytotoxic T cells by expressing higher levels of immunosuppressive molecules, such as PD-L1, ARG1, and IDO, and lower of anti-tumor molecules, such as ROS and TNF-α (37, 38). But the difficulty in distinguishing between N1 and N2 subtypes and mitigating the pro-tumor activities of TANs without affecting their essential immune functions presents challenges in targeting TANs. NES TNBC demonstrates both systemic and local accumulation of neutrophils that have immunosuppressive properties, resulting in resistance to ICB (35, 39). Neutrophils have been found to actuate the reprogramming of macrophage anti-inflammatory by suppressing NF-κB activation, which can balance cytokine generation at a fiery location and influence monocyte and macrophage fiery capacities amid the early stages of aggravation (40). Therefore, neutrophil-to-lymphocyte ratio (NLR) could be examined as a prognostic marker, with higher NLR values associated with worse outcomes (41). It’s mentioned that intratumoral genetic NLR-low TNBC was associated with a favorable tumor immune microenvironment (42). Consequently, exploiting neutrophils in monoclonal antibodies (mAbs) therapy can result in long-term antitumor immunity (43).

Dendritic cells contain a heterogeneous category that play fundamental roles in modulating both innate and adaptive immune responses by functioning as key initiators and regulators (44, 45). There exists considerable interest in the modulation of DC function as a means of enhancing tumor immunotherapy, and various strategies have been formulated to target DCs in tumors. Potential interventions for enhancing immune response involve the administration of immunomodulatory antigens and the advancement of dendritic cell-based vaccines. Numerous categories of vaccines directed towards dendritic cells have been employed in clinical investigations to enhance cancer therapy. The administration of antigens and adjuvants to DCs within the body is a critical approach toward the development of DC vaccines (46–48). Additionally, cytokine-induced killer (CIK/DC-CIK) cell immunotherapy is a therapeutic method that uses *in vitro* expansion and activation of CIK cells to eliminate tumor cells. CIK cells bind to the LFA-1 ligand on tumor cells through the surface adhesion molecule LFA-1, forming effector cell-target cell contacts. They induce tumor apoptosis through the Fas signaling pathway and can secrete a variety of cytokines to regulate the immune microenvironment and enhance anti-tumor immunity (49, 50). Recent clinical trials have been implemented to evaluate the efficacy of DC-CIK immunotherapy in solid tumors such as TNBC setting. The clinical result from SYSUCC demonstrates enhancement in the prognosis of patients with post-mastectomy breast cancer when utilizing adjuvant alternative CIK cell therapy in conjunction with natural killer cell immunotherapy (51). A multi-center national-wide phase II study investigating DC-CIK immunotherapy in 686 pretreated solid tumor patients is also under conduction in SYSUCC (NCT04476641). A personalized vaccine platform using autologous DCs, pulsed with tumor membrane vesicles made from tumor tissue, encapsulating antigens from individual tumors, could also provide the basis for personalized TNBC immunotherapy (46). Challenges such as the efficiency of CIK cell expansion, potential toxicity, and the complexities of personalizing vaccines need to be addressed to improve these therapeutic approaches.

Cancer-associated fibroblasts (CAFs) are a type of stromal cells that play a crucial role in TME of TNBC (52). CAFs promote proliferation, migration, and invasion of tumor cells through G protein-coupled estrogen receptor, and produce immune suppression in BC by inducing lipid-associated macrophages (53–55). TNBC can be classified into two CAF subtypes (CAF+ and CAF-) based on gene expression profiles, while the CAF- subtype was linked to longer overall survival and more immune cells than the CAF+ subtype (56). Understanding the origin and heterogeneity of CAFs is crucial to developing novel strategies targeting pro-tumor CAF subpopulations, which can improve treatment affectability and barrier tumor development. CAF among TNBC microenvironment can be identified into six subpopulations by single-cell RNA sequencing that are generally expressed in antigen-presenting cells, including myofibroblastic CAFs, inflammatory CAFs, and a CAF subpopulation expressing MHC II, which could be targeted as potential therapeutic approaches (52, 57). To date, major CAF-targeting strategies include decreasing CAFs in TME through CAR-T-cell therapy (58), a monoclonal antibody targeting fibroblast activation protein and tumor endothelial marker 8 (59), reducing immunosuppressive functions of CAFs to achieve greater T-cell accessibility to tumor cells and increased sensitivity to therapeutic approaches, decreasing the immunosuppressive characteristics of CAFs so that improving T cell accessibility to tumor cells and increasing sensitivity to therapeutic approaches. Targeting CAFs is challenging due to their heterogeneity and plasticity, which complicates therapy development and risks inadvertently affecting normal fibroblasts and tissue homeostasis. Despite these challenges, reducing CAFs’ immunosuppressive functions to enhance T-cell accessibility to tumor cells remains a promising goal.

### 2.2 Tumor heterogeneity

Tumor cells also demonstrate significant heterogeneity in TNBC, characterized by varying gene copy numbers, mutations, and losses, with somatic copy number alterations (CNAs). Generally, the presence of a germline CNA is typically associated with genomic instability, chromosomal vulnerability, tumor immune suppression, and poorer prognosis (60–62). TNBC can be classified in accord with genomic characteristics, and specific immune subtypes exhibit a strong association with certain CNAs (15, 17, 63, 64). For example, the basal-like 1 subclass of TNBC demonstrates a significantly elevated level of CNAs compared to other TNBC subclasses (65, 66), including the amplifications and gains of MYC, PIK3CA, CDK6, AKT2, KRAS, FGFR1, IGF1R, CCNE1, and CDKN2A/B gene. Conversely, the subtype is also marked by deletions of the BRCA2, PTEN, MDM2, and RB1 genes. The luminal androgen subtype demonstrates a repetitive increase in EGFR and AKT1, while also frequently presenting deletions in CCND3, AKT2, ESR1, CDKN2A/B, SMAD4, NF1, NCOR1, and TP53. While mesenchymal subtype manifests frequent losses of PDGFRA, RB1, and MAP3K1, concomitant with recurrent gains or amplification of DNMT3A and TP53 (65).

Genetic mutations in either somatic or germ cells are indicators of immune response and microenvironment. TP53 mutations may enhance immunogenic activity in breast cancer, indicating TP53 mutation status as a potential biomarker for immunotherapy-responsive breast cancer patients (67, 68). Besides TP53, genetic mutations in BRCA1 and BRCA2 genes can affect the prognosis and treatment options for TNBC (69). Compared to BRCA1/2 wild-type tumors, BRCA1/2 mutation-associated cancers are more immunogenic. Some clinical trials have shown that patients with BRCA-mutant TNBC can achieve better survival and remission rates after receiving immune checkpoint inhibitors combined with chemotherapy or targeted drugs (70; 71). Olaparib combined with Durvalumab has good tolerability and response rate in patients with gBRCA-positive recurrent TNBC (8).

Epigenetic modifications and transcriptional reprogramming play an important role in drug therapy resistance and are considered critical in promoting tumor heterogeneity and TNBC progression (35, 39, 72–76). The characteristics of epigenetic changes mainly include DNA hypermethylation, dysfunction of covalent histone modifications, and chromatin deregulation, which result in TME regulation (77–82). The profiling of DNA methylation in TNBC tumors has revealed additional insights into the DNA methylation signatures that are associated with lymph node metastases and the identification of biomarkers in differentially methylated regions can foresee neoadjuvant therapy outcome (83). Dysregulation of histones covalent modifications is another prominent mechanism that has been demonstrated to be of utmost importance in the process of transcriptomic reprogramming, which plays a role in developed resistance to chemotherapy (84, 123 | Clinical Cancer Research | American Association for Cancer Research, n.d.; 82). Chromatin dysregulation drives TNBC biology via significant transcriptome changes (39, 81, 85). Epigenetic drugs modify cancer and immune cells, enhancing immunity, which could be promising if used combined with immune checkpoint inhibitors. Recent clinical trials investigating the combination of entinostat and atezolizumab (NCT02708680) have shown disappointing results in terms of achieving significant outcomes in PFS and secondary endpoints (86, 87). Despite the potential of epigenetic drugs to enhance immunity by modifying cancer and immune cells, the combination approach with immune checkpoint inhibitors requires further investigation to establish its efficacy in improving patient outcomes.

## 3 Molecular subtype of TNBC in immunotherapy

Currently, the primary recognized strategy for identifying appropriate TNBC patients for ICB are immune scores based on PD-L1 expression levels such as combined positive score (CPS) (88). Pembrolizumab is approved for use in the neoadjuvant setting for all patients with high-risk early-stage TNBC and those with a PD-L1 CPS >10 in the first-line metastatic setting. However, using different clones in immunohistochemistry (IHC) for PD-L1 detection, such as SP142 and 22C3, is important due to their poor consistency in TNBC patients, which can impact patient selection for immunotherapy (89). To address this issue, standardized approaches and further cross-validation between different assays are needed to ensure accurate patient stratification.

In the IMpassion130 trial, insignificant results were detected between the CPS>10 group and CPS>1 group, indicating that immunotherapy for TNBC requires additional selection criteria and biomarkers. The partial failure of CPS in metastases setting could be explained by the developed immune suppression in the heterogeneous microenvironment. This highlights a critical limitation of using PD-L1 as a sole biomarker, as it may not adequately capture the complexity of immune evasion mechanisms in TNBC. Therefore, adopting immune phenotypes and immune identifiers for precise patient selection could be a possible solution to improve the prognosis of immunotherapy. Furthermore, the ongoing combination therapy necessitates the utilization of biomarkers to correspond with individual patients with their optimal treatment alternative (90).

Numerous groups have employed various classifications in the past ten years to subtype TNBC, either by histochemistry, gene expression, mRNA and lncRNA expression, or metabolic pathways (**Table 1**). In 2011, Lehmann’s research took the process of profiling gene expression in tumor samples obtained from a cohort of 587 TNBC patients, which resulted in the delineation of basal like-1 (BL1), basal like-2 (BL2), luminal androgen receptor (LAR), immunomodulatory (IM), mesenchymal (M), mesenchymal stem-like (MSL) subtypes (66). Currently, research primarily focuses on the molecular characteristics of TNBC subtypes, whereas the specific microenvironment features remain unclear and require further investigation.

**Table 1:** Major TNBC stratification methods.

Classification	Subtype	Frequency(%)	Characteristics	Therapeutic Value
TNBCtype-6, 2011	BL1 BL2 IM M MSL LAR	18-26 10-15 10-20 12-20 8-16 10-15	Cell cycle, DNA damage Growth factor signaling Immune signaling Mesenchymal differenciation and proliferation Mesenchymal differenciation with low proliferation Hormone-related	Platinum-based chemotherapy mTOR inhibitors ICIs EMT and CSCs inhibitors EMT and CSCs inhibitors Anti-androgen therapy or CDK4/6 inhibitors
Burstein, 2015	BLIA BLIS LAR MES	49 23 15 13	Immune active, high proliferation Immune suppression, high proliferation Hormone-related Mesenchymal differenciation and proliferation	ICIs Target therapy or PARP inhibitors Anti-androgen therapy or CDK4/6 inhibitors EMT and CSCs inhibitors
TNBCtype-4, 2016	BL1 BL2 M LAR	35 22 27 16	Cell cycle, DNA damage Growth factor signaling Mesenchymal differenciation and proliferation Hormone-related	Platinum-based chemotherapy mTOR inhibitors EMT and CSCs inhibitors Anti-androgen therapy or CDK4/6 inhibitors
FUSCC, 2016	IM LAR MES BLIS	17 18 33 32	Immune signaling Hormone-related Mesenchymal differenciation and proliferation Immune suppression, high proliferation	ICIs Anti-androgen therapy or CDK4/6 inhibitors EMT and CSCs inhibitors Target therapy or PARP inhibitors
Metabolic Pathways, 2020	MPS1 MPS2 MPS3	26 37 37	Lipogenic Glycolytic Mixed phenotype	Lipid synthesis inhibitors LDH inhibitors and ICIs Need to explore

Generally speaking, the basal-like immune-associated (BLIA) subtype in Burstein’s subtyping overlaps with FUSCC IM subtype, which responds promisingly to immunotherapy (92). IM group exhibits immune response-related signatures and high expression levels of checkpoint inhibitory genes, including CTLA-4, PD-1, and PD-L1, which could be identified and suggest promising responses for ICIs (65). LAR subtype, also identified as androgen receptor (AR)-positive tumors and accounting for 10-15% of all TNBC, is characterized by a luminal-like gene expression profile, low proliferation rate, and resistance to chemotherapy. Progressing clinical trials suggest that effective androgen suppressors may improve anti-tumoral activity. The BLIS designates a subtype of TNBC that exhibits a particularly unfavorable prognosis and shows a dearth of immune activation by down-regulating B cell, T cell, and natural killer cell immune-regulating pathways, leading to the conjecture that the administration of ICB is unlikely to confer clinical benefits (91, 93). Several scientific literature has provided evidentiary support for a correlation between basal-like breast cancers and the manifestation of CK5/6, CK14, CK17, P-cadherin, p53, and EGFR. Mesenchymal or mesenchymal stem-like subtype was associated with higher angiogenic signature scores and characteristics of breast cancer stem cells (CSCs) (93, 94). The MES subtype exhibits distinct pathways, including cell cycle, mismatch repair, and DNA damage networks. Therefore, the application of beta-blockers, IGF inhibitors, or PDGFR inhibitors may prove to be promising therapeutic strategies for the management of MES tumors (95). The employment of EZH2-inhibitory agents also represents a promising strategy for reinstating MHC-1 expression in immune cold, PD-1 negative M-subtype tumors (47, 94). Despite their utility, current subtyping strategies and biomarkers have notable limitations, particularly in capturing the complex TNBC microenvironment. These strategies focus on molecular characteristics, often neglecting the tumor microenvironment. Future research should develop integrated approaches that consider both molecular and microenvironmental factors to improve patient stratification and treatment outcomes.

### 3.1 Rational strategies in TNBC immunotherapy

Recent advances in TNBC immunotherapy have brought new hope for improving outcomes in TNBC. A deeper exploration of existing and emerging therapeutic approaches is essential for developing effective treatment strategies. Current research highlights the promise of CAR-T cell therapy, immune checkpoint inhibitors, ICIs combination therapies, and inhibitors targeting specific pathways within the tumor microenvironment. As the field progresses, integrating these diverse therapeutic strategies can significantly improve patient outcomes in TNBC. However, the efficacy of these approaches remains inconsistent and often limited, necessitating a critical evaluation of existing and emerging strategies.

Immune checkpoint inhibitors especially PD-1 inhibitors have demonstrated efficacy by restoring immune function and enabling T cells to attack tumor cells. The sole accepted biomarker to identify TNBC patients benefit from ICIs is the expression of PD-L1, evaluated by the CPS and tumor proportion score (TPS) (13, 96, 97), yet the efficacy is not optimized. TNBC exhibits high levels of PD-L1, which promotes researchers to design multiple TNBC clinical trials using PD-L1 inhibitors thereby discovering the clinical benefits of adding ICB as first-line and second-line therapy for TNBC (98). Currently, the US FDA has approved four PD-L1 reagents and six ICIs, including 22C3 for Pembrolizumab, 28-8 for Nivolumab, SP142 for Atezolizumab, and SP263 for Durvalumab. Despite TNBC’s high PD-L1 expression levels, which have led to numerous clinical trials investigating PD-L1 inhibitors, the effectiveness of CPS as a sole biomarker has been questioned. A significant limitation of CPS as sole biomarker is its variable efficacy in different clinical settings, particularly in metastatic TNBC, where immune suppression within the tumor microenvironment can affect outcomes. Furthermore, the FDA has approved two ICIs, Avelumab and Cemiplimab, that do not show clinical association with PD-L1 expression status (97). This suggests that other biomarkers, such as tumor mutation burden (TMB), tumor-infiltrating lymphocytes, and gene expression profiles, might also play critical roles in predicting patient responses to immunotherapy (99, 100). In this context, the evaluation of co-inhibitory (CI) receptors has emerged as an important consideration to enhance the accuracy of patient selection for ICB therapy. The CI receptors, including CTLA4 and PD1, could have crucial but distinct roles in modulating immune responses, highlighting the complexity of the immune landscape in TNBC and the need for a multi-faceted approach to biomarker development (27). Other CI receptors such as immune cells (IC), TMB, LAG-3, TIM-3, and VISTA play critical roles in immune evasion and could provide additional predictive value for immunotherapy outcomes (101–106). Incorporating these CI evaluations alongside PD-L1 status could lead to more precise and personalized treatment strategies, ultimately improving the prognosis for TNBC patients.

Combining ICIs with novel agents such as antibody-drug conjugates (ADCs) and inhibitors targeting specific pathways within the tumor microenvironment (TME) can enhance the overall anti-tumor response. The integration of ICIs with novel antibody-drug conjugates (ADCs) represents a significant advancement in the treatment of TNBC, as suggested by recent clinical trials, such as MORPHEUS-pan BC and BEGONIA. These combination therapies leverage the immune-activating properties of ICIs with the targeted cytotoxic effects of ADCs, providing a dual approach that not only inhibits tumor growth but also stimulates the immune system to attack cancer cells more effectively (107, 124, 109). Specifically, the atezolizumab and sacituzumab govitecan-hziy (Trodelvy) combination has shown promising results in patients with locally advanced or metastatic TNBC (110.). Furthermore, results from Arm 6 and Arm 7 of the BEGONIA trial indicate that the combination of durvalumab and trastuzumab deruxtecan (T-DXd) or datopotamab deruxtecan (Dato-Dxd) holds significant potential for treating TNBC​ (111). Preliminary results from clinical trials have demonstrated that combining ICIs with novel ADCs can significantly improve overall response rates in TNBC patients. The promising outcomes highlight the potential of such combinations to address the challenges of TNBC treatment, with numerous clinical trials currently underway to further explore their efficacy (**Table 2**). The co-expression of the inhibitory immune checkpoint lymphocyte activation gene-3 (LAG-3) and PD-1 has been observed in exhausted T cells (112), where higher levels of LAG-3 and PD-L1 expression were detected in patients with TNBC (113). Preclinical research indicates that the inhibition of certain pathways within the immune system enhances the ability of CD8 T cells to fight against tumors. The simultaneous blockage of PD-1 and LAG-3 pathways yields a potent outcome. These findings suggest that targeting these immune checkpoints can improve anti-tumor responses. Moreover, the newly released findings of the I-SPY2 clinical trial, which assessed the efficacy of anti-LAG-3 and anti-PD1 treatment in patients with early-stage HER2-negative breast cancer, indicated a projected pathologic complete response rate of 60% for individuals with hormone receptor HR-negative, HER2-negative disease and 37% for those with HR-positive, HER2-negative disease (114). Therefore, a series of dual blockade approaches targeting LAG-3 and PD-1 is currently undergoing clinical evaluation as a potential treatment option for advanced breast cancer (NCT03742349 and NCT03005782), and double antibodies are under evaluation. Opdualag, combining nivolumab with relatlimab, was currently approved by the FDA for melanoma treatment, which may be a potential treatment for TNBC as these two malignancies share similarities in immune therapy (115) The combination of LAG-3 blockades and PD-1 blockade has been proven to be safe and promising in mTNBC, but the exact efficacy still needs large-scale clinical evaluation, and double antibody deserves investigation.

**Table 2:** Ongoing clinical trials of immune checkpoint inhibitor combination therapies in TNBC.

IO Therapy	Combination Therapy	Phase	Setting	Sample Size	Estimated completion	ClinicalTrials.gov
Pembrolizumab	Sacituzumab Govitecan	II	Early stage	25	Dec-26	NCT05675579
Pembrolizumab	Sacituzumab	II	Advanced/ metastatic	110	Apr-29	NCT04468061
Pembrolizumab	Sacituzumab Govitecan	II	Early stage	260	Oct-26	NCT04230109 (NeoSTAR)
Pembrolizumab	Sacituzumab Govitecan	II	Early stage	348	Sep-29	NCT06081244 (ADAPT-TN-III)
Atezolizumab	Sacituzumab Govitecan	II	Early stage/metastatic	40	Dec-37	NCT04434040 (ASPRIA)
Pembrolizumab	Sacituzumab govitecan-hziy	III	Early stage/metastatic	1514	Aug-31	NCT05633654 (ASCENT-05)
Pembrolizumab	Sacituzumab Govitecan-hziy	III	Advanced/ metastatic	440	Feb-27	NCT05382286 (ASCENT-04)
Pembrolizumab	Sacituzumab tirumotecan	III	Early stage	1530	Dec-37	NCT06393374 (MK-2870-012)
Magrolimab	Sacituzumab Govitecan nab-paclitaxel paclitaxel	II	Advanced/ metastatic	92	Jan-25	NCT04958785 (ELEVATE TNBC)
Atezolizumab	Ipatasertib, Paclitaxel, Doxorubicin, Cyclophosphamide	II	Early stage	146	Jan-26	NCT05498896 (BARBICAN)
Durvalumab and Pembrolizumab	Datopotamab Deruxtecan	III	Early stage/metastatic	1728	Aug-30	NCT06112379
Durvalumab	Datopotamab Deruxtecan	III	Early stage/metastatic	1075	Mar-30	NCT05629585 (TROPION-Breast03)
Durvalumab	Datopotamab Deruxtecan	III	Advanced/ metastatic	625	Apr-29	NCT06103864
Pembrolizumab Durvalumab Cemiplimab	AMG 386 MK-2206 T-DM1.	II	Early stage	5000	Dec-31	NCT01042379
Pembrolizumab	Tetrathiomolybdate Capecitabine	II	Early stage/metastatic	204	Jul-34	NCT06134375
PD-1 inhibitor	CAB-ROR2-ADC (BA3021)	I/II	Advanced/ metastatic	420	Dec-25	NCT03504488
Ipilimumab Nivolumab Pembrolizumab Atezolizumab Avelumab Durvalumab Cemiplimab	CP-506	I/II	Advanced/ metastatic	126	May-26	NCT04954599
Atezolizumab	Nab-Paclitaxel	III	Advanced/ metastatic	184	Dec-24	NCT04148911
Nivolumab	MEM-288	I	Advanced/ metastatic	61	Nov-26	NCT05076760
Pembrolizumab	ST-067	I/II	Advanced/ metastatic	316	Jan-25	NCT04787042
Atezolizumab	Cabozantinib	Ib	Advanced/ metastatic	1732	Aug-24	NCT03170960
Pembrolizumab	Bortezomib	I	Advanced/ metastatic	20	Dec-24	NCT04265872
Pembrolizumab	LGK974	I/II	Advanced/ metastatic	429	Oct-25	NCT01351103
Nivolumab	XB002	I	Advanced/ metastatic	573	Oct-24	NCT04925284
Atezolizumab	TT-00420	I/II	Advanced/ metastatic	114	Dec-24	NCT05253053
Pembrolizumab	PVX-410	Ib	Advanced/ metastatic	20	Dec-25	NCT03362060
Pembrolizumab	ZEN003694 Nab-Paclitaxel	Ib	Advanced/ metastatic	57	Dec-25	NCT05422794
Pembrolizumab	Lenvatinib	II	Advanced/ metastatic	590	Dec-24	NCT03797326
Pembrolizumab	Lenvatinib	I	Early stage	12	Jul-26	NCT04427293
Pembrolizumab	SGN-LIV1A	I/II	Advanced/ metastatic	186	Jan-26	NCT03310957
Pembrolizumab	JK08	I/II	Advanced/ metastatic	263	Feb-26	NCT05620134
Pembrolizumab	KFA115	I	Advanced/ metastatic	180	Feb-26	NCT05544929
Pembrolizumab	ASTX660	I	Advanced/ metastatic	48	Mar-26	NCT05082259 (ASTEROID)
Pembrolizumab	AN0025	Ib	Advanced/ metastatic	63	Jan-25	NCT04432857
Pembrolizumab	Azenosertib Carboplatin	I/II	Advanced/ metastatic	78	Mar-27	NCT06351332
Pembrolizumab	Olinvacimab	II	Advanced/ metastatic	30	Aug-26	NCT04986852
Pembrolizumab	Capecitabine Talazoparib Inavolisib	II	Early stage/metastatic	197	Jan-34	NCT04849364
Pembrolizumab	INBRX-106	II	Early stage	12	Jun-29	NCT06353997
Pembrolizumab	XmAb808	I	Advanced/ metastatic	220	Dec-27	NCT05585034
Pembrolizumab	Enfortumab vedotin	II	Advanced/ metastatic	320	Sep-26	NCT04225117
Pembrolizumab	Olaparib, Carboplatin, Gemcitabine	II/III	Advanced/ metastatic	462	Nov-25	NCT04191135 (KEYLYNK-009)
Pembrolizumab	Carboplatin Paclitaxel Doxorubicin Epirubicin Cyclophosphamide Filgrastim or Pegfilgastrim	III	Early stage	1174	Sep-25	NCT03036488 (KEYNOTE-522)
Pembrolizumab	Paclitaxel Carboplatin Doxorubicin Cyclophosphamide Capecitabine Olaparib	II	Early stage	30	Jun-30	NCT06245889
Pembrolizumab	Capecitabine Carboplatin Epirubicin Cyclophosphamide Paclitaxel	III	Early stage	920	Jun-35	NCT04335669
Nivolumab	Doxorubicin Cyclophosphamide Cisplatin	II	Advanced/ metastatic	84	Aug-25	NCT02499367 (TONIC)
Atezolizumab	Capecitabine	II	Early stage	284	Jan-27	NCT03756298
Ociperlimab Tislelizumab	Paclitaxel	II	Advanced/ metastatic	/	Jun-29	NCT05809895 (AdvanTIG-211)
Atezolizumab	Paclitaxel Carboplatin Epirubicin Cyclophosphamide	II	Early stage	461	Aug-24	NCT04770272
Pembrolizumab	Olaparib	II	Early stage	23	Jan-26	NCT05203445
Pembrolizumab	Paclitaxel	II	Early stage	354	Dec-31	NCT06078384 (ETNA)
Atezolizumab	Paclitaxel, Carboplatin, Doxorubicin, Cyclophosphamide, Epirubicin1	III	Early stage	1550	Nov-27	NCT03281954
Pembrolizumab	Liposomal Irinotecan (Nal-IRI)	II	Advanced/ metastatic	/	Jan-30	NCT05255666
Pembrolizumab	Carboplatin Gemcitabine	II	Advanced/ metastatic	87	May-26	NCT02755272
Pembrolizumab	Carboplatin Olaparib	II	Early stage	23	Sep-27	NCT05485766
Pembrolizumab	Paclitaxel Carboplatin Cyclophosphamide Docetaxel Doxorubicin	III	Early stage	2400	Mar-33	NCT05929768
Atezolizumab	Gemcitabine and Carboplatin or Capecitabine	III	Advanced/ metastatic	572	Aug-24	NCT03371017 (IMpassion132)
Pembrolizumab	Cisplatin Nab-paclitaxel Olaparib	II	Advanced/ metastatic	136	Mar-25	NCT05174832
Pembrolizumab	Carboplatin Paclitaxel	II	Early stage	28	Sep-27	NCT06318897
Atezolizumab	Nab-paclitaxel	II	Early stage/metastatic	37	Dec-25	NCT02530489
Atezolizumab	Carboplatin Cyclophosphamide Paclitaxel	IIb	Advanced/ metastatic	304	Dec-30	NCT01898117 (Triple-B)
Pembrolizumab	HMBD-002	I/II	Advanced/ metastatic	240	Jan-25	NCT05082610
Nivolumab	Romidepsin Cisplatin	I/II	Advanced/ metastatic	51	Jul-27	NCT02393794
Pembrolizumab	Carboplatin Docetaxel Doxorubicin Cyclophosphamide	II	Early stage	139	Dec-25	NCT05645380 (NeoTRACT)
Nivolumab	Cisplatin doxorubicin	II	Advanced/ metastatic	52	Dec-26	NCT04159818 (TONIC-2)
Pembrolizumab	Docetaxel IL-12 gene therapy	II	Advanced/ metastatic	30	Dec-24	NCT04095689
Durvalumab Tremelimumab	Nab-paclitaxel Neoantigen Vaccine	II	Advanced/ metastatic	70	Dec-24	NCT03606967
Tiragolumab with Atezolizumab	Ipilimumab	II	Advanced/ metastatic	60	Apr-30	NCT06342037 (TONIC-3)
Pembrolizumab	Tavokinogene Telseplasmid	II	Advanced/ metastatic	65	Sep-24	NCT03567720 (KEYNOTE-890)
Pembrolizumab	Paclitaxel Doxorubicin Cyclophosphamide IRX-2	II	Early stage	12	Jun-25	NCT04373031
Pembrolizumab Ipilimumab	TMV vaccine	I	Early stage/metastatic	18	May-33	NCT06324240
Pembrolizumab Ipilimumab	Anti-HER2/HER3 Dendritic Cell Vaccin	II/III	Advanced/ metastatic	23	Dec-25	NCT04348747
Pembrolizumab	ADG106	I/II	Advanced/ metastatic	51	Dec-26	NCT05491083
Atezolizumab	Bevacizumab, Carboplatin, Gemcitabine	II	Advanced/ metastatic	31	Sep-25	NCT04739670 (BELLA)
Pembrolizumab	Cryoablation	I	Advanced/ metastatic	30	Jan-27	NCT06246968
Pembrolizumab	CyPep-1	I/II	Advanced/ metastatic	90	Feb-25	NCT05383170
Pembrolizumab	BT-001 (Oncolytic Vaccinia virus)	I/II	Advanced/ metastatic	48	Apr-25	NCT04725331
Pembrolizumab	SO-C101	Ib	Advanced/ metastatic	200	Nov-24	NCT04234113
Pembrolizumab	Rintatolimod Celecoxib Interferon Alpha 2b	I/II	Advanced/ metastatic	12	Jun-25	NCT05756166
Atezolizumab	Autogene Cevumeran (RO7198457)	I/II	Advanced/ metastatic	272	Nov-24	NCT03289962
Pembrolizumab	ST-alpha-DC1	II	Advanced/ metastatic	19	Oct-26	NCT05539365
Atezolizumab	KY1044	I/II	Advanced/ metastatic	280	Aug-24	NCT03829501
Pembrolizumab	TTX-080	I	Advanced/ metastatic	240	Jun-24	NCT04485013
Pembrolizumab	NM1F (Anti-PVRIG)	I	Advanced/ metastatic	38	Sep-27	NCT05746897
FAZ053 PDR001	FAZ053 PDR001	I	Advanced/ metastatic	154	Nov-25	NCT02936102
Pembrolizumab	TJ107	II	Advanced/ metastatic	133	Dec-24	NCT05145907
Pembrolizumab	NT-I7	I/II	Advanced/ metastatic	215	Mar-25	NCT04332653
Spartalizumab	DKY709	I/Ib	Advanced/ metastatic	98	Dec-24	NCT03891953
Pembrolizumab	PeptiCRAd-1	I	Early stage/metastatic	15	Jan-25	NCT05492682 (START)
Pembrolizumab	MDNA11	I/II	Advanced/ metastatic	115	Dec-26	NCT05086692
Atezolizumab	RP1 Oncolytic Immunotherapy	I/II	Early stage	51	Apr-31	NCT06067061 (neoBREASTIM)
Pembrolizumab	AE37 peptide vaccine.	II	Advanced/ metastatic	29	Jun-24	NCT04024800
Pembrolizumab	Trilaciclib Gemcitabine Carboplatin	II	Advanced/ metastatic	36	Mar-27	NCT06027268 (ToPCourT)
Nivolumab	BMS-986449	I/II	Advanced/ metastatic	100	Jul-27	NCT05888831
Atezolizumab	IPI-549 (eganelisib)	II	Advanced/ metastatic	167	Mar-28	NCT06052852
Nivolumab	BT5528	I/II	Advanced/ metastatic	288	Dec-24	NCT04180371
Nivolumab	LN-145	II	Advanced/ metastatic	30	Jun-25	NCT03449108
Pembrolizumab	BAY3375968	I	Advanced/ metastatic	270	Jun-25	NCT03449108
Pembrolizumab	SGN-PDL1V	I	Advanced/ metastatic	322	Dec-26	NCT05208762

Molecular subtype-based optimized treatment strategies offer a promising outlook for improving therapeutic outcome in TNBC. Utilizing PD-L1 and PD-1 inhibitors has proven efficient in TNBC immunomodulatory subtype, both early-stage and metastases setting. Based on current TNBC subtypes, individuals diagnosed with TNBC are suggested to undergo preliminary screening to evaluate the expression of PD1 or PD-L1 before contemplating the administration of immunotherapy as a treatment modality. ICIs are advisable if the value of CPS surpasses a threshold of 10 in metastatic TNBC and in all patients in the neoadjuvant setting regardless of CPS score, while additional biomarkers such as LAG-3, TILs may also provide additional therapeutic perspective. As an alternative, it is suggested to perform patient testing to assess the existence of androgen receptors and in the event of positive outcomes, it is supported to pursue pharmacological intervention through anti-androgen receptor therapy. Otherwise, it should be noted that the patient may display indications that are congruous with BLIS or MES categories, and the utilization of DNA profiling may have the ability to differentiate among the subcategories and enable the recognition of ideal pharmaceutical treatments. By categorizing TNBC into molecular subtypes such as PD-L1, LAR, BLIS, and MES, treatment strategies could be tailored more effectively, thereby enhancing therapeutic outcomes. The FUTURE-SUPER trial underscores the clinical advantages of employing molecular subtype-based treatment optimization for patients with TNBC, indicating a direction for further clinical research (116). A series of clinical trials have been conducted based on the subtyping of triple-negative breast cancer (**Table 3**).

**Table 3:** Ongoing clinical trials based on TNBC subtypes.

Drugs	Target Pathway	Phase	Setting	Sample Size	Estimated completion	ClinicalTrials.gov
Immunotherapy						
Camrelizumab Famitinib	PD-1 VEGF	II	Advanced/ metastatic	139	Dec-24	NCT04395989 (FUTURE-SUPER)
Camrelizumab Famitinib	PD-1 VEGF	II	Advanced/ metastatic	46	Jan-21	NCT04129996 (FUTURE-C-PLUS)
AZD6738 Olaparib Durvalumab	ATR PARP PD-L1	II	Early stage	81	Dec-25	NCT03740893 (PHOENIX)
Atezolizumab Ipatasertib SGN-LIV1A Bevacizumab Selicrelumab Tocilizumab Sacituzumab Govitecan	PD-L1 AKT LIV-1 VEGF IL-6 IL-6 Trop-2	Ib/II	Advanced/ metastatic	133	May-23	NCT03424005 (Morpheus-TNBC)
Pembrolizumab	PD-1	II	Advanced/ metastatic	160	Feb-26	NCT05852691
Proleukin	IL-2	I/II	Advanced/ metastatic	10	Apr-23	NCT05821686
Pembrolizumab	PD-1	III	Post- neoadjuva nt with PCR	1295	May-33	NCT05812807 (Optimal-PCR)
Ociperlimab Tislelizumab Pembrolizumab	TIGIT PD-L1 PD-L1	II	Advanced/ metastatic	250	Jul-23	NCT05809895 (AdvanTIG-211)
Pembrolizumab	PD-L1	II	Response- adapted	139	Dec-25	NCT05645380 (NeoTRACT)
Ceralasertib Durvalumab	ATR PD-L1	II	Advanced/ metastatic	37	Nov-25	NCT05582538 (ATRiBRAVE)
ST-alpha-DC1 Pembrolizumab	DCs	II	Advanced/ metastatic	19	May-25	NCT05539365
Pembrolizumab Axatilimab	PD-L1 CSF-1R	II	Advanced/ metastatic	35	Dec-24	NCT05491226
CyPep-1 Pembrolizumab	CytC PD-L1	I/II	Advanced/ metastatic	90	Feb-25	NCT05383170
Balstilmab	PD-1	I/II	Advanced/ metastatic	41	Oct-26	NCT05318469
Atezolizumab Ipatasertib Bevacizumab Pertuzumab Trastuzumab	PD-L1 AKT VEGF HER2	II	Early stage	210	Feb-25	NCT05180006
Trilaciclib	CDK4/6	II	Early stage	24	Mar-23	NCT05112536
Choline SHR1210 Efavirenz	ChAT PD-1 NNRTI	II	Advanced/ metastatic	30	Mar-23	NCT05076682
Sintilimab Anlotinib	PD-1 Tyrosine kinase	II	Early stage	46	Dec-25	NCT04877821 (NeoSACT)
Enobosarm Exemestane	SARM P450	III	Advanced/ metastatic	210	Jul-23	NCT04869943 (ARTEST)
Talazoparib Atezolizumab Inavolisib	PARP PD-L1 PI3K/AKT	II	Post-neoadjuvant	197	Jan-34	NCT04849364
Niraparib Dostarlimab	PARP PD-1	II	Advanced/ metastatic	32	Dec-29	NCT04837209
Spartalizumab	PD-1	II	Advanced/ metastatic	73	Dec-24	NCT04802876 (ACROPOLI)
Trilaciclib	CDK4/6	III	Advanced/ metastatic	194	Oct-24	NCT04799249 (PRESERVE 2)
IRX 2 Pembrolizumab	TILs PD-L1	II	Neoadjuvant	12	Jun-25	NCT04373031
IMC-F106C Atezolizumab pembrolizumab	PRAME PD-L1	I/II	Advanced/ metastatic	170	Feb-26	NCT04262466
Avelumab	PD-L1	II	Advanced/ metastatic	150	Jul-23	NCT03971409
Atezolizumab BDB001	PD-L1 TLR7/8	II	Advanced/ metastatic	247	Mar-25	NCT03915678
Ipilimumab Nivolumab	CTLA-4 PD-1	II	Early stage	80	Jan-27	NCT03815890
Oleclumab	CD73	I/II	Advanced/ metastatic	129	Oct-23	NCT03616886 (SYNERGY)
Ipilimuma Nivolumab	CTLA-4 PD-1	II	Early stage	80	Jun-26	NCT03546686
Pembrolizumab	PD-L1	III	Adjuvant	1155	May-26	NCT02954874
Pembrolizumab Binimetinib	PD-L1 MEK	I/II	Advanced/ metastatic	38	Jul-23	NCT03106415
Atezolizumab	PD-L1	II	Early stage	72	Jul-23	NCT02883062
Atezolizumab	PD-L1	II	Adjuvant	37	Feb-23	NCT02530489
CR1447	ERα	II	Advanced/ metastatic	29	Jun-27	NCT02067741
Androgen positive TNBC						
Ceralasertib Durvalumab	ATR PD-L1	II	Advanced/ metastatic	37	Nov-25	NCT05582538
Everolimus Pyrotinib	mTOR PI3K/AKT	II	Advanced/ metastatic	139	Dec-24	NCT04395989 (FUTURE-SUPER)
EP0062	HER2 EGFR	I/II	Advanced/ metastatic	128	Mar-25	NCT05573126
Abemaciclib Bicalutamide	CDK4/6 AR	I/II	Advanced/ metastatic	60	Sep-24	NCT05095207
Enobosarm Abemaciclib Everolimus	AR CDK4/6 mTOR	III	Advanced/ metastatic	186	Jan-24	NCT05065411
Seviteronel-D	AR	Ib	Advanced/ metastatic	65	Dec-24	NCT04947189
Enobosarm Exemestane	AR ER	III	Advanced/ metastatic	210	Jul-23	NCT04869943
Enzalutamide	AR	II	Early stage	37	Dec-23	NCT02689427
Palbocicilib Bicalutamide	CDK4/6 AR	I/II	Advanced/ metastatic	46	Nov-24	NCT02605486
Enzalutamide	AR	II	Advanced/ metastatic	118	Dec-23	NCT01889238
BLIS/MES TNBC						
Bevacizumab	VEGFR	II	Advanced/ metastatic	139	Dec-24	NCT04395989 (FUTURE-SUPER)
BP102	VEGFR	II	Advanced/ metastatic	192	26-Feb	NCT05806060
VP-16	VEGFR	I/II	Advanced/ metastatic	140	22-Dec	NCT03805399 (FUTURE)

In recent years, CAR-T cell therapy has made significant progress in the treatment of solid tumors, including TNBC. Historically, the immunosuppressive signals within the TME of solid tumors have limited the efficacy of CAR-T cells. In TNBC, research is ongoing to enhance the delivery of tumors and improve the persistence of CAR-T cells. In preclinical studies and early clinical trials, several antigens have been established as viable targets for CAR-T cell therapy in TNBC. NKG2D ligands, expressed on various tumor types and immunosuppressive cells within the tumor microenvironment, present a promising target for cancer therapy (117). In mouse studies, CAR-T cells engineered with derivatives of HLA-A2/NY-ESO-1 have been used in cancer immunotherapy, showing extended overall survival in TNBC and primary melanoma models (125). However, the selection of optimal targets remains a challenge in CAR-T cell therapy to minimize off-target effects and enhance specificity (118). Early results from clinical studies show that CAR-T therapy in TNBC has not led to significant on-target, off-tumor toxicities related to specific targets like ROR1 (119). Clinical and preclinical models have identified numerous antigens suitable for CAR-T cell therapy in TNBC. TROP2, GD2, ROR1, MUC1, and EpCAM have been identified as the most promising targets, and CARs developed against these targets have shown the ability to penetrate and migrate through TNBC cultures, eliciting significant antitumor responses (108, 118, 120–122). To summarize, the successful application of CAR-T cell therapy in TNBC requires overcoming barriers related to the tumor microenvironment and antigen heterogeneity.

In summary, while immunotherapy offers significant promise for TNBC treatment, its current application is hampered by variability in patient response, the need for better biomarkers, and the challenges associated with advanced therapeutic strategies. Future research should focus on optimizing these approaches, improving patient selection criteria, and developing more effective and less toxic combination therapies (99, 100).

## 4 Discussion

### 4.1 Heterogeneity and subtyping of TNBC

Triple-negative breast cancer is a heterogeneous group containing several distinct subtypes. Subtyping TNBC is deemed necessary to properly identify suitable patients for immunotherapy as well as facilitate the identification of optimal alternate treatment protocols for non-responsive patients. TNBC can be classified into different molecular subtypes based on gene expression profiles. Different profiles within each category demonstrate the distinct characteristics of the immune response, metabolism processes, and supporting tissue. Nonetheless, most previous clinical studies have not focused on distinct subpopulations to identify efficacy indicators. Recent research has identified biomarkers for characterizing TNBC subtypes and assessing therapeutic effects of drugs, filling a critical research gap.

### 4.2 Tumor immune microenvironment

TNBC contains a diverse TME that includes TILs, macrophages, neutrophils, DCs, and CAFs. These components can impact tumor growth and the immune response, and each of them holds prognostic value and potential for targeted therapy. TNBC exhibits high immunogenicity through TILs, which are associated with clinical outcomes. TILs can serve as a subtyping biomarker for immune response in TNBC, and personalized TILs immunotherapy shows promise for patients with drug-resistant TNBC. Targeting the macrophage-enriched subtype and reprogramming macrophages from an immunosuppressive M2 phenotype to a pro-inflammatory state can enhance the effectiveness of ICIs. TANs, particularly the immunosuppressive N2 subtype, contribute to ICB resistance; modulating TANs and using the NLR as a prognostic marker can help tailor therapeutic approaches and improve outcomes. Dendritic cell-based therapies, such as vaccines and CIK cell immunotherapy, are being explored to enhance anti-tumor immune responses, including the administration of immunomodulatory antigens and the use of autologous DCs pulsed with tumor antigens for personalized immunotherapy. Targeting specific CAF subpopulations and reducing their immunosuppressive functions can increase T cell accessibility to tumor cells and improve sensitivity to therapies; strategies include CAR-T-cell therapy and monoclonal antibodies targeting CAF-associated markers. To improve the response to immunotherapy, it is crucial to classify TNBC based on TME characteristics and consider combining TME-targeted therapies. Regular monitoring of TME changes using biomarkers can help adjust treatment plans and serve as prognostic indicators. For instance, adopting immune phenotypes and immune identifiers for precise patient selection could improve immunotherapy prognosis. Combining TME-targeted treatments with immunotherapy could address the issue of immunosuppression in the tumor environment, potentially leading to better outcomes.

### 4.3 Ethical considerations in TNBC immunotherapy

Ethical considerations are crucial in the development and application of TNBC immunotherapy. The potential for off-target effects, where therapies inadvertently impact non-cancerous tissues, poses significant risks to patients. For instance, immune checkpoint inhibitors can trigger severe immune-related adverse events affecting organs such as the liver, lungs, and endocrine glands. Ensuring thorough preclinical testing and vigilant monitoring during clinical trials can mitigate these risks. Furthermore, the need for informed consent is paramount, as patients must be fully aware of the potential benefits and risks associated with new treatments. Transparent communication about the experimental nature of some therapies and the possibility of adverse effects is essential for ethical clinical practice. Furthermore, equitable access to these novel treatments and considering the socioeconomic factors that may influence patient participation in clinical trials are critical ethical issues.

### 4.4 Advancements in TNBC management

The management of TNBC is undergoing substantial changes, as the identification and characterization of the distinct molecular profile of the tumors, including the evaluation of PD-L1 and the androgen receptor, are broadening the spectrum of therapeutic interventions available in clinical practice. It’s promising for future research agendas to focus on the identification of additional targetable and innovative biomarkers, which have the potential to define therapeutic targets or prognostic indicators more comprehensively. The treatment of TNBC has evolved beyond a uniform application for all individuals, and subgroup therapeutic regimens are anticipated. Additional clinical trials are being conducted to demonstrate the efficacy of new medications and assess the potential benefits of identifying novel biomarkers. These advancements pave the way for more precise and personalized treatment strategies, ultimately aiming to improve long-term outcomes for patients with TNBC. To further improve the efficacy of TNBC immunotherapy, it is crucial to delve deeper into several key areas. Identifying and validating additional biomarkers for patient stratification and predicting response to immunotherapy is essential. In-depth studies on molecular immune subtyping of TNBC subtypes and clinical research exploring prognosis can enhance treatment strategies. Exploring combination therapies that target multiple components of the TME may provide a more comprehensive approach to overcoming tumor resistance. Personalized immunotherapies based on the unique genetic and immune profiles of individual tumors will allow for more effective treatments. Investigating the mechanisms of resistance to immunotherapy and strategies to overcome them is critical for improving long-term outcomes for patients with TNBC.

## Nomenclature

### Resource identification initiative

To take part in the Resource Identification Initiative, please use the corresponding catalog number and RRID in your current manuscript. For more information about the project and for steps on how to search for an RRID, please click here.

### Life science identifiers

Life Science Identifiers (LSIDs) for ZOOBANK registered names or nomenclatural acts should be listed in the manuscript before the keywords with the following format:

urn:lsid:<Authority>:<Namespace>:<ObjectID>[:<Version>]
